# Epigenetic and Endocrine Adaptations Linking Chronic Pain, Metabolic Dysregulation, and Cardiovascular Remodeling: A Narrative Review

**DOI:** 10.7759/cureus.100757

**Published:** 2026-01-04

**Authors:** Oyebisi M Azeez, Happiness Olaniyi, Enobong Obong, Caleb C Dunkwu, Japheth O Oyovwi, Mary I Oyovwi, Saerimam N Markus, Chinaecherem Peace Okafor, Oluwatobiloba K Adedokun, Aliyu O Olaniyi

**Affiliations:** 1 Veterinary Physiology and Biochemistry, University of Ilorin, Ilorin, NGA; 2 Nursing, Barton Brook Care Home, Manchester, GBR; 3 Neurology, Washington University School of Medicine, St. Louis, USA; 4 Internal Medicine, Calderdale and Huddersfield NHS Foundation Trust, Huddersfield, GBR; 5 Geriatrics, Stepping Hill Hospital, Manchester, GBR; 6 General Practice, Bolton NHS Foundation Trust, Manchester, GBR; 7 Biology, The University of Alabama at Birmingham, Birmingham, USA; 8 Biochemistry, The Graduate Center, City University of New York (CUNY), New York, USA; 9 Surgery, General Hospital Odan Lagos, Lagos, NGA

**Keywords:** cardiovascular remodeling, chronic pain, endocrine dysregulation, epigenetics, inflammation, metabolic syndrome

## Abstract

Chronic pain is not confined to the areas of nociceptive input but activates systemic biological reactions characterized by inflammation, endocrine disequilibrium, and new epigenetic remodeling. The combination of these processes leads to cardiovascular dysfunction and metabolic dysregulation, but there is limited understanding of the integrative processes between them. The available evidence indicates that maladaptive changes in the vascular and metabolic systems are accompanied by sustained neuroendocrine and epigenetic reprogramming and require a one-stop synthesis of information.

This narrative review will explain the role of epigenetic and endocrine mediators of the interaction between chronic pain, cardiovascular remodeling, and metabolic dysfunction, and shed light on important understanding of mechanisms and implications of translation.

The literature search was performed in PubMed, Scopus, and Web of Science databases, with the keywords related to chronic pain, epigenetics, endocrine adaptation, cardiovascular remodeling, and metabolic health. Both animal and human studies in the English language were incorporated. Synthesis of evidence was done in a narrative fashion with a focus on mechanistic themes, although limited by the fact that heterogeneity of study designs was noted and the possibility of selection bias in narrative reviews was also indicated.

Chronic pain has been associated with activation of the hypothalamic-pituitary-adrenal (HPA) axis and the sympathetic-adrenal axis, which may contribute to sustained elevations in cortisol and catecholamines, insulin resistance, and endothelial dysfunction. Evidence from animal models and observational human studies suggests that epigenetic mechanisms, including DNA methylation, histone modifications, and microRNA regulation, are involved in modulating inflammatory and vascular responses, potentially favoring maladaptive gene expression linked to myocardial hypertrophy, vascular constriction, and metabolic syndrome. Moreover, bidirectional interactions between hormonal signaling and epigenetic regulation are thought to exacerbate oxidative stress, inflammation, and metabolic dysregulation, forming a reinforcing loop that may help sustain elevated cardiometabolic risk.

The modulation of chronic pain is associated with sustained endocrine and epigenetic restructuring, including chronic activation of the hypothalamic-pituitary-adrenal axis with altered cortisol and catecholamine signaling, alongside epigenetic modifications of stress- and vascular-related genes involved in inflammation, endothelial function, and metabolic regulation (e.g., *NR3C1* and endothelial nitric oxide synthase). These changes may contribute to cardiovascular remodeling and dysmetabolic states. Accordingly, pain management should be integrated with cardiometabolic risk assessment to provide holistic patient care. Future mechanistic and longitudinal studies should be prioritized to clarify causal pathways and to identify therapeutic targets capable of mitigating the systemic effects of chronic pain.

## Introduction and background

Chronic pain is one of the most prevalent and burdensome health conditions worldwide, affecting an estimated 20%-30% of the global population and accounting for a substantial proportion of disability, reduced quality of life, and healthcare expenditure [1]. Clinically and in research settings, chronic pain is defined as pain persisting for ≥3 months beyond the expected period of tissue healing, in accordance with International Association for the Study of Pain criteria, and encompasses nociceptive, neuropathic, inflammatory, and mixed pain phenotypes [1]. Unlike acute pain, which serves a protective physiological function, chronic pain persists beyond normal healing and evolves into a complex biopsychosocial condition [2]. It arises from diverse and often overlapping etiologies, including musculoskeletal, neuropathic, inflammatory, and idiopathic causes, and is frequently accompanied by systemic comorbidities [3].

While chronic pain is frequently discussed as a unified clinical entity, the systemic biological effects described in this review do not apply uniformly across all pain etiologies. Evidence linking chronic pain to sustained inflammation, endocrine dysregulation, and cardiometabolic risk is strongest in conditions characterized by persistent inflammatory activity and/or pronounced central sensitization, such as fibromyalgia, chronic widespread pain, inflammatory arthritis, and neuropathic pain syndromes. In contrast, purely localized nociceptive pain appears less consistently associated with systemic alterations unless it progresses to a centrally amplified or persistent state. Accordingly, much of the mechanistic and epidemiological evidence discussed herein is driven by these centrally mediated and inflammatory pain conditions, a distinction that is emphasized throughout the review [2]. Increasing evidence indicates that chronic pain is not merely a localized sensory experience but a systemic condition characterized by dysregulation of neuroimmune, endocrine, and metabolic pathways, with important implications for long-term cardiometabolic health.

Continuous pain conditions trigger inflammatory pathways in which circulating levels of cytokines such as interleukin (IL)-1β and IL-6 and tumor necrosis factor-alpha (TNF-alpha) are raised and have detrimental effects on vascular endothelium, autonomic, and myocardial integrity [4]. This is accompanied by a persistent activation of the hypothalamic-pituitary-adrenal (HPA) axis and sympathetic nervous system that causes hormonal dysregulations, such as cortisol imbalance and excess secretion of catecholamines [5]. Such endocrine changes serve to augment hypertension, dysfunction of endothelial cells, insulin resistance, and dyslipidemia, markers of cardiovascular and metabolic pathology. Therefore, chronic pain has become a possible cause of cardiometabolic disease development and progression of hypertension, atherosclerosis, obesity, and type 2 diabetes mellitus [6,7].

The concept of a pain-induced “molecular memory” refers to the persistence of epigenetic modifications that outlast the initiating nociceptive stimulus. In recent years, attention has increasingly focused on epigenetic reprogramming as a central integrative mechanism driving the multisystem effects of chronic pain, whereby long-term changes in gene expression are mediated through DNA methylation, histone modifications, and regulation by non-coding RNAs [8]. Chronic pain-related inflammation and neuroendocrine stress can remodel epigenetic landscapes across immune, vascular, and metabolic tissues, promoting maladaptive immune activation, altered vascular gene expression, and disrupted metabolic signaling [9]. These sustained epigenetic changes provide a biological basis for a long-lasting molecular memory within immune and endocrine systems, which perpetuates inflammatory, endocrine, and cardiovascular remodeling processes even after the original pain stimulus has resolved [10]. However, evidence supporting this concept derives primarily from experimental and preclinical models, while human data remain largely indirect and cross-sectional; longitudinal studies confirming the stability and clinical relevance of such epigenetic signatures following chronic pain exposure are still lacking [1].

Studies in epidemiology and clinical practice are showing a growing association between chronic pain and increased rates of cardiovascular dysfunction and metabolic disorders [11]. Cardiovascular risk profiles, such as an increase in arterial stiffness, autonomic imbalance, and dyslipidemia, can be high in patients with chronic back pain, fibromyalgia, or neuropathic pain [12]. In addition, comorbidity with metabolic syndrome or diabetes is seen in relation to a worse outcome, implying that chronic pain and these diseases are shared pathways, which lie outside the standard disease boundaries. Although these had been noticed, the mechanistic perspective of the effects of pain on systemic cardiometabolic dysregulation has not been fully understood.

Endocrine and epigenetic adaptation seems to be key intermediates between chronic pain and cardiovascular and metabolic remodeling. With constant endocrine stress responses, epigenetic regulations of inflammatory and metabolic activity might be altered, and conversely, epigenetic changes during pain can impair hormonal homeostasis. These bidirectional communications are probably involved in the long-term vascular and metabolic effects of chronic discomfort, providing targets of treatment intervention. Invariably, epigenetic regulators most consistently linked to cardiometabolic outcomes include DNA methylation of *NR3C1* and eNOS, and microRNAs such as miR-21, miR-155, and miR-146a, as well as pathways related to inflammatory signaling, endothelial dysfunction, and metabolic regulation [13,14].

Epidemiological associations between chronic pain and cardiometabolic disease are derived predominantly from cross-sectional and observational cohort studies, with relatively few longitudinal investigations. Although several cohorts suggest that chronic pain predicts future cardiovascular morbidity independent of traditional risk factors, causal inference remains limited by residual confounding and restricted long-term follow-up [1,2]. In light of the growing recognition of chronic pain as a multisystem disorder, this narrative review synthesizes existing evidence on the endocrine and epigenetic adaptive mechanisms that may underlie the observed links between chronic pain, metabolic dysregulation, and cardiovascular remodeling. In doing so, it aims to highlight key knowledge gaps, summarize emerging mechanistic insights, and propose future research directions to inform preventive and therapeutic strategies addressing the systemic consequences of chronic pain.

## Review

Methods

This narrative review was done in an attempt to synthesize and contextualize available evidence appraising chronic pain as epigenetic and endocrine actions on cardiovascular remodeling and metabolic dysregulation. Three large electronic databases, namely, PubMed, Scopus, and the Web of Science Core Collection, were searched to have a comprehensive literature search. To enable the search of both established and emerging evidence in the field, the search strategy was limited to studies published between January 2000 and October 2025.

The search employed combinations of the following keywords and Medical Subject Headings (MeSH): “chronic pain,” “epigenetics,” “DNA methylation,” “histone modification,” “microRNA,” “endocrine dysregulation,” “HPA axis,” “cardiovascular remodeling,” “metabolic syndrome,” “insulin resistance,” and “systemic inflammation.” Boolean operators (AND, OR) were applied to refine the search and identify studies relevant to the interconnected biological pathways among chronic pain, endocrine alterations, and cardiometabolic outcomes.

The inclusion criteria were original research, reviews, meta-analyses, and experimental or clinical studies in English that measured the relationship between chronic pain and epigenetic or endocrine adaptations, with the preference being those that measured cardiovascular or metabolic endpoints. Both animal and human research were seen to include mechanistic and translational comprehensiveness. Conference abstracts, editorials, case reports, and those studies that lacked explicit mechanistic or systemic outcome information formed the exclusion criteria.

Titles and abstracts were used first in screening the articles, after which articles that qualified according to the inclusion criteria were evaluated for their data. The major conclusion was classified into major themes, including (1) chronic pain as a systemic disorder, (2) epigenetic changes, (3) endocrine changes, (4) the interdisciplinary mechanisms that bind these systems, and (5) clinical and translational outcomes.

As this review is narrative in nature, no quantitative synthesis or meta-analysis was done. Rather, the qualitative integration of the findings was done to give conceptual unity and also bring out the emergent trends in the mechanisms. The review emphasizes the biological plausibility, consistency of evidence, and broad translational relevance but limited clinical specificity, rather than statistical generalizability.

The possible methodological weaknesses are publication bias, differences in study design, and heterogeneity of the outcome measures across the literature. In addition, the narrative synthesis methodology does not allow the formal assessment of risks of bias. Nevertheless, the review gives more priority to high-quality peer-reviewed research and cross-verifies across a variety of sources so as to have a balanced and evidence-based discussion.

Chronic pain as a systemic disorder

Chronic pain is increasingly recognized as a multidimensional condition that extends far beyond persistent sensory nociception. Conventionally defined as pain lasting longer than three months, chronic pain may be classified by etiology as nociceptive, neuropathic, inflammatory, or mixed [15]. Nociceptive pain arises from tissue injury and inflammation, whereas neuropathic pain results from damage to the somatosensory nervous system. Importantly, maladaptive neuroplastic changes and the development of central sensitization enable pain to persist even in the absence of ongoing tissue damage, underscoring the complex neurobiology of chronic pain [16,17]. Central sensitization provides a critical mechanistic bridge between persistent pain and systemic immune and endocrine dysregulation. Sustained amplification of nociceptive signaling within spinal and supraspinal networks engages hypothalamic and brainstem nuclei that regulate autonomic outflow and hypothalamic-pituitary-adrenal (HPA) axis activity, resulting in prolonged exposure to glucocorticoids and catecholamines. These neuroendocrine signals directly alter immune cell transcriptional programs, cytokine production, and epigenetic regulation of stress-responsive genes, thereby actively driving neuroimmune maladaptation rather than representing a mere epiphenomenon of chronic pain.

Consistent with this framework, chronic pain is associated with generalized physiological alterations, including a state of low-grade systemic inflammation characterized by persistently elevated pro-inflammatory cytokines such as interleukin-6 (IL-6), tumor necrosis factor-α (TNF-α), and interleukin-1β (IL-1β). While peripheral inflammatory sources such as injured tissues and activated immune cells contribute to increased circulating cytokine levels, accumulating evidence indicates that centrally mediated mechanisms dominate once pain becomes chronic. Persistent activation of central stress-regulatory circuits, particularly the HPA axis and the sympathetic nervous system, amplifies immune signaling through neuroimmune communication and hormonal modulation of immune cell function. Thus, systemic inflammation in chronic pain reflects not only peripheral nociceptive input but also centrally driven stress-immune dysregulation [18,19].

In parallel with neuroendocrine activation, chronic pain induces a persistent autonomic nervous system imbalance characterized by sympathetic overactivation and parasympathetic withdrawal. This dysautonomia contributes to elevated blood pressure, peripheral vasoconstriction, increased heart rate variability, and reduced baroreflex sensitivity. Sustained activation of the sympathetic-adrenal-medullary system not only increases cardiovascular load but also modulates immune and metabolic function, thereby amplifying inflammatory signaling and physiological stress responses [20,21]. Concurrently, pain-related psychological and physiological stress chronically stimulates the hypothalamic-pituitary-adrenal (HPA) axis, leading to sustained elevations in circulating cortisol. While acute cortisol release is adaptive, prolonged exposure disrupts glucose homeostasis, inhibits endothelial nitric oxide production, and promotes vascular stiffening. Together with inflammatory neuroimmune communication, these endocrine adaptations converge on endothelial dysfunction, a critical early event in the pathogenesis of cardiovascular disease [5,22].

Epidemiological evidence consistently demonstrates an association between chronic pain and increased cardiometabolic disease burden. Conditions such as hypertension, coronary artery disease, and metabolic syndrome occur at higher prevalence among individuals with fibromyalgia, osteoarthritis, and neuropathic pain compared with pain-free populations [23,24]. These associations likely reflect a combination of direct biological effects and partial mediation by behavioral factors, including reduced physical activity, sleep disturbance, depression, and medication use. Importantly, several large observational and longitudinal studies report that chronic pain independently predicts cardiovascular morbidity even after adjustment for these variables, suggesting that cardiometabolic risk arises through both behavioral and pain-specific biological pathways. Mechanistically, this relationship is mediated by a convergence of endothelial injury driven by systemic inflammation, autonomic dysregulation, and hormonal imbalance, alongside maladaptive behavioral factors that further exacerbate cardiometabolic vulnerability [25].

Importantly, available evidence indicates that autonomic imbalance and endothelial dysfunction associated with chronic pain may be at least partially reversible, particularly in earlier disease stages and with effective pain control, stress reduction, and lifestyle interventions. However, prolonged exposure to neuroendocrine and inflammatory stress appears to promote progressive vascular and myocardial remodeling, a process more clearly demonstrated in experimental models and long-standing clinical cohorts. Longitudinal human studies directly examining reversibility versus irreversible progression remain limited. Collectively, these findings support the conceptualization of chronic pain as a systemic pathophysiological condition rather than a localized symptom. Its sustained inflammatory and neuroendocrine activation initiates a cascade of vascular, metabolic, and immunological disturbances that predispose to cardiovascular remodeling and metabolic dysfunction, providing the framework for examining how endocrine and epigenetic adaptations may perpetuate these adverse health outcomes in subsequent sections of this review [26].

Epigenetic modifications in chronic pain and systemic dysregulation

Epigenetic mechanisms are emphasized in this review because they serve as dynamic integrators of endocrine, inflammatory, and environmental signals, linking sustained pain-related stress to stable yet potentially reversible changes in gene expression. Unlike genetic variation, which is static, or behavioral factors, which are distal and heterogeneous, epigenetic regulation provides a biologically plausible interface through which chronic inflammation and neuroendocrine stress responses can drive coordinated cardiometabolic alterations [4]. Epigenetic pathways enable environmental and physiological stimuli to induce long-lasting modifications in gene expression without altering the underlying DNA sequence, primarily through DNA methylation, post-translational histone modifications, and non-coding RNA regulation, particularly microRNAs, that shape cellular phenotypes across immune, neural, vascular, and metabolic systems [27,28]. In the context of chronic pain, persistent nociceptive input, inflammation, and endocrine stressors converge to remodel epigenetic landscapes, sustaining maladaptive inflammatory programs, altering vascular gene expression, and disrupting metabolic signaling in ways that promote cardiovascular remodeling and metabolic dysfunction [14].

DNA methylation predominantly occurs at cytosine residues within CpG dinucleotides and is commonly associated with transcriptional repression when present at gene promoters. Persistent exposures to chronic inflammatory and stressful stimuli have the potential to cause hyper- or hypomethylation of loci controlling immune- and endocrine-related responses [29]. For example, alterations of gene expression of the glucocorticoid signaling pathway (i.e., the glucocorticoid receptor) have been observed in stress-related disease and are likely to change the sensitivity of the HPA axis during chronic pain disease state [30]. Equally, the pro-inflammatory or dysfunctional vascular phenotype can be entrained by changes in methylation of promoters of cytokine expression (or endothelial homeostasis, such as regulators of endothelial nitric oxide synthase) and may stimulate cellular dysfunction, defective vasodilation, and atherogenesis [31].

Transcriptional activity of target genes depends on histone modifications, such as acetylation, methylation, phosphorylation, and ubiquitination, which control the accessibility of the chromatin [32]. Histone acetylation is mostly linked with transcriptional activation, and certain methylation patterns can either activate or repress transcription in a context-dependent manner [33]. Changes in histone acetylation and methylation in preclinical models of persistent nociception and inflammatory disease have been linked to the upregulation of pro-inflammatory gene networks and the expression of matrix remodeling and fibrotic genes in cardiac and vascular tissue [34]. Such histone modifications might thus support transcriptional programs in favor of vascular stiffness, extracellular matrix deposition, and myocardial remodeling, all of which are cardiovascular pathological characteristics of chronic pain cohorts [35].

MicroRNAs, non-coding short RNAs that both hinder translation of target mRNAs and trigger degradation, offer cell-type and rapid post-transcriptional control. Some of the microRNAs that have been found to be involved in inflammatory signaling and cardiovascular biology are also deregulated in the pain and stress paradigms. An example of such is microRNA-155, a pro-inflammatory regulator that increases the production of cytokines in immune cells; its expression stimulates systemic inflammation and, thus, adds to the vascular injury. MicroRNA-21 has been pointed out to be involved in fibroproliferative reactions and cardiac fibrosis, and is also regulated by inflammatory and neuroendocrine signals. MicroRNA-146a, conversely, has often been found to be a negative-feedback mechanism of innate immune signaling; downregulation of its expression may constitute a break on inflammation. Intuitive to these and to other microRNA networks, a pain-induced, epigenetic response can enhance inflammatory production and, at the same time, regulate endothelial, myocardial, and metabolic cell behavior [36-38].

The effects of epigenetic remodeling in chronic pain on the cell- and tissue-based functions result in a set of convergent pathophysiological themes that pertain to cardiometabolic health. Epigenetic reprogramming has the potential to maintain an elevated pro-inflammatory phenotype in immune cells, which keeps the vasculature and myocardium under ongoing cytokine-induced stress. Endothelial activation, the recruitment of leukocytes, and the dysfunction of microvessels in endothelial cells are favored by the epigenomic silencing of endothelial gene-supportive genes of nitric oxide generation and anti-adhesive phenotypes. Histone and microRNA modifications in cardiomyocytes and cardiac fibroblasts may stimulate hypertrophic and fibrotic transcriptional programs, which may result in structural remodeling and dysfunctional cardiac performance. Epigenetic changes occurring in adipocytes and hepatocytes in metabolic tissues contribute to adipokine release, insulin signals, and lipid metabolism, those underlying mechanisms that in turn increase vascular disease [14,39].

Preclinical and clinical studies collectively support the role of epigenetic mechanisms in linking chronic pain, stress responses, and downstream cardiovascular and inflammatory sequelae. In animal models of chronic inflammatory or neuropathic pain, reproducible epigenetic alterations have been observed in the dorsal root ganglia, spinal cord, and stress-responsive brain regions, with progressive involvement of peripheral tissues including the vasculature and myocardium [40]. Experimental manipulation of these epigenetic marks, such as through histone deacetylase inhibitors or microRNA mimics and antagonists, attenuates nociceptive behaviors, as well as subsequent inflammatory or fibrotic remodeling, supporting a causal role in disease propagation [14].

Human evidence, while more limited, is steadily expanding. In cohorts with persistent pain, stress-related disorders, or chronic inflammatory disease, analyses of peripheral blood and accessible tissues reveal differential DNA methylation and microRNA expression profiles that correlate with pain severity, systemic inflammation, metabolic dysfunction, and markers of subclinical cardiovascular disease [40,41]. Notably, the most consistently replicated findings in human studies involve altered DNA methylation of stress- and inflammation-related genes, particularly *NR3C1* (encoding the glucocorticoid receptor), and reproducible changes in circulating microRNAs, such as miR-21, miR-155, and miR-146a. In contrast, evidence for histone modifications within neural, vascular, or myocardial tissues, as well as epigenetic regulation of fibrotic and hypertrophic gene programs, is largely derived from preclinical pain and stress models. Explicitly distinguishing these evidence tiers is essential to appropriately weight translational confidence [39]. In humans, evidence for pain-associated DNA methylation changes remains largely cross-sectional. For example, altered methylation of stress-responsive genes such as *NR3C1* has been reported in peripheral blood from individuals with chronic pain and stress-related disorders, correlating with pain severity and dysregulated HPA axis signaling; however, these findings are derived predominantly from observational cohorts rather than studies on epigenetic and endocrine adaptation with longitudinal designs. Notably, well-powered longitudinal human studies demonstrating the temporal stability or reversibility of pain-associated epigenetic signatures are still scarce [14].

In interpreting the current literature, hypothesized causal mechanisms are therefore primarily supported by experimental and longitudinal animal studies, whereas human data largely reflect associative relationships derived from cross-sectional analyses. This limitation underscores the need for well-designed prospective and interventional studies to establish temporal relationships, clarify causality, and guide translational strategies targeting the endocrine-epigenetic axis in chronic pain [41]. Additional challenges to clinical translation include the dynamic and potentially reversible nature of epigenetic marks, which makes them attractive therapeutic targets, and also their sensitivity to age, sex, genotype, diet, smoking, and medication exposure, complicating causal inference. Moreover, many existing studies are constrained by small sample sizes, heterogeneous tissue sampling, and variability in epigenetic measurement techniques [42].

Overall, epigenetic pathways represent biologically plausible and growingly validated mechanisms by which chronic pain results could cause long-term molecular markings on immune, vascular, cardiac, and metabolic systems. Such imprints aid in giving reasons as to how persistent nociceptive and neuroendocrine stressors are transformed to long-term inflammatory, endothelial dysfunction, myocardial remodeling, and metabolic dysregulation, connections discussed further in the ensuing sections [14].

Endocrine adaptations to persistent pain

Repeated nociceptive input and the psychological stress accompanying chronic pain drive sustained activation of central stress systems, particularly the hypothalamic-pituitary-adrenal (HPA) axis and the sympathetic-adrenal-medullary system. While acute activation of these pathways constitutes an adaptive response that supports cardiovascular homeostasis under transient stress, chronic pain is characterized by persistent nociceptive and psychological stress that results in maladaptive stress physiology. Prolonged stimulation increases corticotropin-releasing hormone and adrenocorticotropic hormone secretion, leading to sustained elevations in circulating glucocorticoids alongside increased catecholamine release from sympathetic nerve endings and the adrenal medulla [5,43]. Over time, this chronic activation produces maladaptive endocrine phenotypes, including glucocorticoid receptor desensitization, flattened diurnal cortisol rhythms, chronic hypercortisolemia, and sustained catecholaminergic tone [44]. Concomitantly, autonomic imbalance marked by sympathetic predominance and parasympathetic withdrawal amplifies these endocrine disturbances, providing a mechanistic link between persistent pain and downstream cardiovascular remodeling [5,6].

Sustained elevations in cortisol and catecholamines exert widespread effects on cardiovascular and metabolic systems. Cortisol promotes hepatic gluconeogenesis, reduces insulin sensitivity in peripheral tissues, facilitates visceral adiposity, and enhances vascular smooth muscle reactivity, sodium retention, and arterial stiffness [45,46]. Excess catecholamines increase heart rate and myocardial oxygen demand, activate hypertrophic signaling pathways, exacerbate endothelial dysfunction through oxidative stress, and reduce nitric oxide bioavailability. These endocrine perturbations further interact with metabolic regulators, including impaired pancreatic β-cell function, altered adipokine profiles, and activation of the renin-angiotensin-aldosterone system, collectively driving insulin resistance, vascular fibrosis, and structural cardiac remodeling. Importantly, these chronic alterations, rather than short-term stress responses, are most relevant to the development of long-term cardiovascular and metabolic risk in individuals with chronic pain, forming a mechanistic interface between persistent pain and cardiometabolic dysfunction (Table 1) [47].

**Table 1:** Summary of Key Epigenetic and Endocrine Mechanisms Linking Chronic Pain to Cardiometabolic Dysregulation HPA: hypothalamic-pituitary-adrenal, HDAC: histone deacetylase

Mechanistic domain	Molecular mediators	Physiological impact	Clinical implications
Epigenetic modifications	DNA methylation (e.g., NR3C1, eNOS), histone acetylation, microRNAs (miR-21, miR-155, miR-146a)	Sustained inflammatory gene expression, endothelial dysfunction, vascular remodeling	Potential biomarkers for pain-induced cardiovascular risk; therapeutic targets for epigenetic modulation [48]
Endocrine dysregulation	Chronic HPA axis activation (↑ cortisol), sympathetic overdrive (↑ catecholamines), altered adipokines (↑ leptin, ↓ adiponectin)	Insulin resistance, hypertension, visceral adiposity, myocardial hypertrophy	Targets for stress-axis modulation (e.g., β-blockers, behavioral therapy) [49]
Cross-talk pathways	Glucocorticoid receptor-histone interaction, redox-sensitive HDAC activity, cytokine-driven microRNA regulation	Feed-forward loop between inflammation, oxidative stress, and hormonal imbalance	Integrative biomarker panels combining endocrine and epigenetic readouts [50]
Cardiometabolic outcomes	Endothelial dysfunction, vascular stiffness, myocardial fibrosis, insulin resistance, dyslipidemia	Elevated cardiometabolic disease risk in chronic pain populations	Supports multidisciplinary screening and prevention strategies [51]

Cross-talk between epigenetic and endocrine pathways

Endocrine cues and epigenetic machinery engage in reciprocal cross-talk that can initiate and perpetuate maladaptive systemic responses in chronic pain states. Glucocorticoids and catecholamines influence gene expression not only through classical receptor-mediated transcriptional pathways but also via direct regulation of epigenetic modifiers. For example, activation of the glucocorticoid receptor can recruit histone-modifying enzymes and chromatin-remodeling complexes, thereby altering histone acetylation and methylation patterns and progressively attenuating transcriptional responsiveness at stress- and immune-related loci. Prolonged glucocorticoid exposure is additionally associated with changes in DNA methylation across inflammatory and stress-responsive genes, leading to altered hypothalamic-pituitary-adrenal (HPA) axis feedback set points and dysregulated immune activation. Similarly, adrenergic signaling can modulate the activity of DNA methyltransferases and histone deacetylases, reshaping chromatin accessibility within vascular and immune cells and reinforcing pro-inflammatory and metabolic gene programs [14,30].

**Figure 1:**
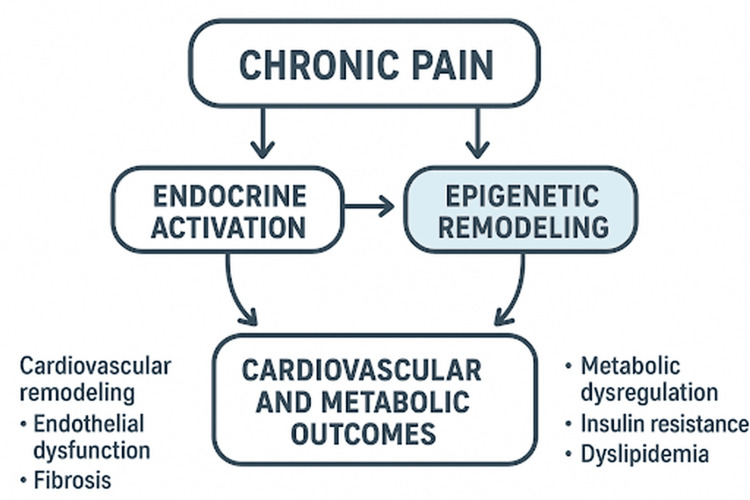
Conceptual Model of Chronic Pain, Induced Endocrine Activation, and Epigenetic Remodeling in Cardiovascular and Metabolic Dysfunction Source: The figure was created by the authors. Figure description: Solid arrows denote pathways supported by human observational and experimental evidence, whereas dashed arrows indicate hypothesized or predominantly preclinical mechanisms. Feedback loops represent self-reinforcing interactions between endocrine dysregulation, epigenetic modification, and systemic inflammation. Associations between chronic pain and endocrine/inflammatory alterations are well supported in human studies, while epigenetic regulation of vascular, myocardial, and metabolic remodeling is derived largely from preclinical data.

Figure 1 illustrates a theoretical model of the endocrine epigenetic axis in chronic pain. Persistent nociceptive input activates the HPA axis and the sympathetic-adrenal system, resulting in sustained elevations of cortisol and catecholamines. These hormonal signals induce epigenetic modifications, including DNA methylation changes, histone remodeling, and microRNA imbalances, which stabilize inflammatory and metabolic dysfunction. In turn, coordinated endocrine and epigenetic alterations promote cardiovascular remodeling and metabolic dysregulation, establishing a self-reinforcing feedback loop that may contribute to long-term disease progression.

A key interpretive challenge in this framework is distinguishing epigenetic alterations directly driven by chronic pain exposure from those secondary to comorbid stress, systemic inflammation, obesity, or prolonged pharmacologic treatment. Evidence from pain-specific cohorts and experimental pain models indicates that sustained nociceptive signaling and central sensitization can independently reshape epigenetic regulation of stress response and immune genes. Nevertheless, glucocorticoid excess, inflammatory cytokines, and metabolic disturbances frequently present in chronic pain populations also exert powerful epigenetic effects. Consequently, many observed epigenetic signatures likely reflect convergent and interacting influences rather than pain-specific mechanisms alone, underscoring the need for carefully phenotyped, longitudinal, and interventional studies to disentangle causality and refine translational targeting of the endocrine epigenetic axis.

In return, the sensitivity and production of endocrine systems change with epigenetic changes. Receptor density and signaling efficacy can be modified by DNA methylation or histone modification of promoters of hormone receptor genes (i.e., glucocorticoid receptor or alpha-adrenergic receptor genes) and, in this way, determine endocrine responsiveness to stress. Inflammatory and hormonal regulation of microRNAs also act as an extra cross-regulation level: microRNA tracks regulated by hormones can target transcripts of proximal signal mediators, epigenetic regulating enzymes, or metabolic regulators, whereas the expression of the microRNAs is influenced by epigenetic marks and transcription factors dependent on endocrine status [30].

This cross-talk of different molecules culminates in various integrated responses that maintain inflammation, oxidative stress, and vascular remodeling. Epigenetic modification (driven by hormones) can permanently commit immune cells to a pro-inflammatory phenotype, which permanently sustains NF-nimbus activity and cytokine expression even with no external agony. Prolonged exposure to cytokines and endocrine imbalances promotes mitochondrial dysfunction and the generation of reactive oxygen species, as well as additional alterations of epigenetic enzymes, such as redox-sensitive regulation of histone deacetylases, creating feedback loops of oxidative stress and chromatin remodeling. In vascular and myocardial cells, these processes upregulate profibrotic pathways (e.g., transforming growth factor-β) and downregulate genes that maintain endothelial nitric oxide synthase activity and anti-inflammatory programs. These molecular changes act as switches that drive smooth muscle hyperplasia, extracellular matrix deposition, and myocardial fibrosis [10].

Collectively, the mutual regulation between endocrine signaling and epigenetic regions can offer a believable mechanistic theory of the mechanisms through which transient or persistent pain may induce long-lasting modifications in vascular organization or metabolic governance. The interactions further indicate that the points of intervention can involve epigenetic modifiers, normalizing endocrine dysregulation or disrupting critical nodes of hormone-epigenome-immune cross-talk, which can reverse or suppress pain-related cardiometabolic remodeling. The study needs longitudinal mechanistic experiments, which combine hormonal profiling with epigenomic mapping in the pertinent tissues to un-cause causality to define the specific acts of therapeutic therapy.

Cardiovascular remodeling and metabolic consequences

With respect to temporal sequencing, current evidence does not permit definitive conclusions as to whether epigenetic alterations precede cardiometabolic dysfunction or arise as downstream adaptations to established disease. Experimental pain and stress models indicate that epigenetic reprogramming can occur early, contributing causally to inflammatory, metabolic, vascular, and myocardial changes. In contrast, human studies typically assess epigenetic marks contemporaneously with overt cardiometabolic abnormalities, thereby limiting causal inference. Collectively, these observations support a bidirectional framework in which epigenetic alterations may both initiate and perpetuate cardiometabolic pathology over time [40].

Within this framework, the convergence of chronic pain-induced endocrine and epigenetic perturbations activates molecular programs that drive structural and functional remodeling of the heart and vasculature while simultaneously promoting adverse metabolic phenotypes. Sustained activation of neurohormonal systems, including catecholaminergic signaling, glucocorticoid excess, and the renin-angiotensin-aldosterone system, triggers intracellular hypertrophic pathways within cardiomyocytes [52]. These signaling cascades induce hypertrophy-associated gene transcription and stimulate cardiac fibroblast proliferation with increased extracellular matrix deposition [53]. Such maladaptive remodeling is further reinforced by epigenetic mechanisms, including altered histone acetylation patterns and disease-associated microRNA expression, particularly the upregulation of profibrotic microRNAs that promote myocardial fibrosis and reduce ventricular compliance. The resulting cardiac phenotype commonly manifests as concentric or mixed hypertrophy, impaired diastolic function, and heightened susceptibility to heart failure with preserved or reduced ejection fraction, depending on the prevailing hemodynamic load, metabolic stress, and inflammatory milieu [54]. In this context, epigenetic modifications may act both as early mediators of endocrine-driven remodeling and as stabilizing mechanisms that entrench pathological gene expression programs once cardiometabolic disease is established.

In the vasculature, interactions among inflammatory cytokines, oxidative stress, and endocrine dysregulation promote endothelial dysfunction and vascular stiffening. Cytokine-mediated inhibition of endothelial nitric oxide synthase, coupled with oxidative inactivation of nitric oxide, impairs vasodilatory capacity and predisposes to increased peripheral resistance. Epigenetic repression of genes involved in endothelial maintenance and repair further compromises vascular resilience, fostering chronic endothelial activation, leukocyte adhesion, and a prothrombotic state. Concurrently, catecholamine- and growth factor-exposed vascular smooth muscle cells undergo phenotypic switching toward a synthetic, proliferative state, a process amplified by epigenetic regulation of matrix-remodeling enzymes such as matrix metalloproteinases. Collectively, these alterations drive progressive arterial stiffening, increased pulse wave velocity, and impaired coronary microvascular function, features frequently observed in individuals with chronic inflammation or persistent pain [55,56].

Throughout this section, it is important to distinguish correlational evidence derived primarily from cross-sectional and observational human studies from mechanistic inferences that are supported largely by experimental and longitudinal animal models. While human data consistently demonstrate associations between chronic pain and adverse cardiometabolic profiles, the causal pathways described here are inferred from biological plausibility and preclinical experimentation rather than being conclusively established in clinical populations.

These cardiac and vascular changes have both direct and indirect mechanisms of their metabolic effects. Endocrine changes, which include glucocorticoid hyperactivity and sympathetic hyperactivity, stimulate hepatic gluconeogenesis, lipid catabolism accompanied by lipid ectopic deposition, and visceral adipose cell overgrowth. The epigenetic remodeling of adipose tissue, including the change of methylation of genes regulating adipokine synthesis, may inhibit adiponectin synthesis and enhance leptin resistance, which further causes insulin resistance and chronic low-grade inflammation. Hyperglycemia and hyperinsulinemia are caused by impaired skeletal muscle glucose uptake caused by interference of insulin receptor signaling by inflammatory cytokines. These processes are often accompanied by dyslipidemia, i.e., hepatic insulin resistance changes the very-low-density lipoprotein metabolism, and systemic inflammation changes the functionality of high-density lipoprotein (HDL), making HDL less cardioprotective [46,57]. Together, these endocrine and epigenetic changes favor the classic symptoms of metabolic syndrome: central obesity, high fasting glucose, hypertension, and atherogenic dyslipidemia, each of which triggers a feedback mechanism of aggravated vascular and cardiac remodeling.

These mechanistic connections are also supported by the empirical evidence of links found in the preclinical and clinical worlds. Chronic stress or chronic pain animal models display cardiac hypertrophy, augmented deposition of collagen, endothelial malfunction, and a diminished glucose tolerance, as well as measurable epigenetic changes in the cardiac and metabolic tissues. The presence of increased prevalence of insulin resistance, central adiposity, and dyslipidemia in groups with chronic pain diagnosis has been identified in human observational studies with annual studies in biomarkers showing the simultaneous increase of IL-6, C-reactive protein (CRP), and fluctuation of cortisol rhythms. Subclinical cardiovascular biomarkers, including increased carotid intima thickness, diminished flow-mediated dilation, and augmented arterial stiffness, are more prevalent in populations with chronic pain and are associated with inflammatory and endocrine biomarkers. Although their direct longitudinal association with pain-induced epigenetic signatures has yet to be found, the cumulative value of mechanistic and associative evidence points to a model where chronic pain makes cardiometabolic risks happen swiftly along endocrine and epigenetic pathways, which are interlaced [58,59].

Clinical and translational implications

The identification of the endocrine-epigenetic axis in pain-related cardiometabolic disease offers several translational opportunities for biomarker development, targeted therapies, and focused research. While human observational studies consistently support associations between chronic pain, systemic inflammation, autonomic imbalance, HPA axis dysregulation, and increased cardiometabolic risk, tissue-specific epigenetic mechanisms and direct causal links to structural cardiovascular remodeling remain largely inferred from experimental models. Accordingly, the proposed endocrine-epigenetic feedback loop should be interpreted as a biologically plausible integrative framework rather than definitive evidence of causality in clinical populations.

From a translational perspective, potential biomarker panels may include inflammatory markers (e.g., IL-6, TNF-α, and CRP), endocrine markers such as salivary or serum cortisol dynamics, plasma catecholamines, fasting insulin, DNA methylation signatures of stress- and inflammation-related loci (from blood or accessible tissues), and circulating microRNAs including miR-21, miR-155, and miR-146a [60]. Multi-marker approaches integrating these domains have the potential to improve risk stratification for cardiovascular remodeling and metabolic deterioration in chronic pain populations and may serve as intermediate outcome measures in future interventional studies rather than population-level screening tools [61].

Therapeutically, interventions can be conceptualized across three overlapping levels: anti-inflammatory modulation, normalization of endocrine stress responses, and targeted epigenetic mechanisms. Anti-inflammatory strategies include lifestyle interventions such as exercise, weight control, and dietary modification, alongside pharmacologic approaches including nonsteroidal anti-inflammatory drugs and, in selected cases, biologic agents targeting cytokines such as IL-6 or IL-1 [62]. Endocrine-oriented interventions encompass behavioral stress management, cognitive behavioral therapy, and pharmacologic modulation of sympathetic tone or HPA axis activity (e.g., β-blockers, selective sympatholytics, or circadian cortisol rhythm restoration) [63]. At the epigenetic level, preclinical evidence supports the potential of histone deacetylase inhibitors, DNA methyltransferase modulators, and microRNA-based therapies to reverse maladaptive gene expression programs; however, clinical translation remains unproven, with unresolved challenges related to safety, tissue specificity, and long-term effects [64].

Importantly, available evidence suggests that analgesia alone may be insufficient to fully mitigate long-term cardiometabolic risk, particularly in individuals with prolonged pain exposure and established systemic dysregulation [65]. The greatest clinical benefit is therefore likely to arise from integrated care models that combine effective pain management with proactive cardiometabolic risk assessment and systemic intervention. Routine screening for hypertension, dyslipidemia, impaired glucose tolerance, and subclinical vascular disease in chronic pain populations, ideally guided by validated biomarkers, may facilitate earlier prevention of downstream cardiovascular events. An individualized approach that accounts for sex differences, age, genetic background, and comorbidities is essential, given heterogeneity in pain phenotypes and systemic responses [66,67].

Limitations

Human evidence most consistently links chronic pain to systemic pathways including low-grade inflammation, autonomic imbalance, HPA axis dysregulation, endothelial dysfunction, and insulin resistance, while tissue-specific epigenetic changes and direct causal links to cardiovascular remodeling are largely inferred from experimental models, underscoring both translational relevance and evidentiary limitations. Major gaps persist due to the scarcity of longitudinal human studies defining the timing, tissue specificity, and adaptive versus maladaptive nature of cardiometabolic remodeling across pain phenotypes, compounded by the narrative review design and lack of formal reproducibility measures. No interventional trials have yet tested whether targeting inflammatory, hormonal, or epigenetic mechanisms can prevent or reverse cardiovascular changes in chronic pain, and interpretation is further complicated by confounding factors such as age, sex, medication exposure, lifestyle behaviors, obesity, sleep disturbance, and socioeconomic stress. An additional forward-looking limitation is that the manuscript does not yet provide guidance on which tissues (e.g., blood, adipose, muscle, or vascular tissue) or which epigenetic assays (e.g., targeted DNA methylation or miRNA profiling) are most feasible or informative for future longitudinal studies, highlighting the need for adequately powered, sex-stratified investigations that integrate clearly defined tissue selection with endocrine, inflammatory, and epigenomic profiling to clarify causal pathways and inform therapeutic strategies.

## Conclusions

Chronic pain is increasingly recognized as a systemic disorder that extends beyond sensory perception, inducing long-term endocrine and epigenetic adaptations with broad physiological consequences. These interactions promote cardiovascular remodeling, including endothelial dysfunction, vascular activation, and myocardial hypertrophy, while simultaneously contributing to metabolic dysregulation, such as insulin resistance, obesity, and dyslipidemia. A convergence of inflammatory, hormonal, and epigenetic signals creates a self-reinforcing loop that perpetuates cardiometabolic susceptibility, even after the initial nociceptive stimulus has resolved.

To manage this complicated interplay, a multidisciplinary approach to a clinical framework that incorporates pain management along with early cardiometabolic risk detection and interventions is needed. This should be a combination of efficient analgesia, stress management, and lifestyle change alongside active examination of cardiovascular and metabolic diseases, such as developing epigenetic and endocrine biomarkers. The next crucial step in the field lies in systematic, mechanistic, and translational studies that can be used to unwind the cause and effect, in addition to establishing the ability to reverse causal action on the molecular targets and the effectiveness of interventions that alter both the experience of pain and its systemic manifestations. By bridging neuroscience, endocrinology, and cardiovascular medicine, these efforts could position chronic pain management at the heart of cardiometabolic prevention.
